# An Interactive Preoperative Virtual Reality Intervention for Breast Cancer Patients Undergoing Oncological Surgery: A Feasibility and Pilot Randomized Clinical Trial

**DOI:** 10.1002/jso.70269

**Published:** 2026-04-23

**Authors:** Renée El‐Gabalawy, Gabrielle S. Logan, Pamela Hebbard, Jordana L. Sommer, Kristin Reynolds, Kailey E. Penner, Michael S. D. Smith, Natalie Mota, W Alan C. Mutch, Eisi Mollanji, Jessica L. Maples‐Keller, David Perrin, Rakesh C. Arora

**Affiliations:** ^1^ Department of Clinical Health Psychology University of Manitoba Winnipeg Manitoba Canada; ^2^ Department of Anesthesiology, Perioperative and Pain Medicine University of Manitoba Winnipeg Manitoba Canada; ^3^ Department of Psychology University of Manitoba Winnipeg Manitoba Canada; ^4^ Department of Psychiatry University of Manitoba Winnipeg Manitoba Canada; ^5^ CancerCare Manitoba Winnipeg Manitoba Canada; ^6^ Department of Surgery University of Manitoba Winnipeg Manitoba Canada; ^7^ National Research Council Canada Winnipeg Manitoba Canada; ^8^ Kleysen Institute for Advanced Medicine (KIAM) Winnipeg Manitoba Canada; ^9^ Department of Psychiatry and Behavioral Sciences Emory University Atlanta Georgia USA; ^10^ Department of Surgery Northwestern University Chicago Illinois USA

**Keywords:** breast cancer, oncology, perioperative, virtual reality, VR

## Abstract

**Background and Objectives:**

Preoperative anxiety is common before surgery and is associated with adverse outcomes, yet access to mental health support remains limited. We evaluated the feasibility and acceptability of a novel preoperative virtual reality (VR) prototype designed to reduce anxiety in patients undergoing cancer surgery.

**Methods:**

In a multi‐method feasibility and pilot trial, participants were randomized to either the VR intervention or standard of care (SoC) between 2021 and 2023. VR participants completed a simulation 1–2 weeks before surgery exploring the operating room (OR) and experiencing anesthesia induction. Intervention feasibility and acceptability (primary outcomes), and preliminary trends in anxiety and distress (secondary outcomes) were explored using measures and open‐ended questions administered at four perioperative timepoints.

**Results:**

Of 33 interested individuals, 27 were randomized, and 23 completed the study (VR: *n* = 12; SoC: *n* = 11). No adverse VR effects occurred. Most participants reported elevated preoperative anxiety (78.3%–87%), rated the VR as helpful (M = 80.4%), enjoyable (M = 87.7%), and all viewed it as worthwhile. Six qualitative themes emerged: preparation for the OR, psychological and emotional impact, realism, interactivity, technical issues, and supporting others. Trends suggested reduced postoperative distress and anxiety for the VR group.

**Conclusion:**

The VR intervention was feasible and acceptable, supporting further development and advancement to a larger clinical trial.

**Trial Registration**: The clinical trial was registered at clinicaltrials.gov: https://clinicaltrials.gov/study/NCT04544618 on September 10th, 2020.

## 1 Introduction

Preoperative anxiety and distress are frequently described as the worst component of the perioperative experience [1], and are associated with adverse perioperative outcomes, including complications, poor physical functioning and pain, extended hospital stay, and some mixed findings on mortality [2, 3, 4, 5, 6, 7]. Amplified by the uncertainty surrounding unpredictable treatment courses [8, 9], approximately 30% of patients undergoing oncological surgeries experience clinically significant rates of perioperative distress [10, 11]. Specifically, for breast cancer surgery, distress typically peaks before surgery and sharply decreases afterwards [12], highlighting that this distress is linked to the anticipation of surgery itself.

Preoperative exposure to the operating room (OR) has been associated with decreased anxiety in cancer surgery patients through familiarization with an often‐unfamiliar and intimidating environment, facilitating security and safety [13]. Recent research supports the use of preoperative psychological interventions to improve outcomes in cancer surgery patients [14]. However, mental health resources are limited, and interventions such as OR tours are not routinely feasible in practice, warranting innovations in treatment implementation.

Virtual reality (VR) is increasingly being used to deliver psychological interventions perioperatively. Two recent meta‐analyses concluded that VR interventions were associated with reduced preoperative anxiety in surgical patients, with VR exposure to surgical settings showing better efficacy than VR distraction approaches (e.g., games [15, 16]). Indeed, one recent systematic review demonstrated the promise and effectiveness of VR OR tours in alleviating perioperative patient anxiety [17]. The literature on using VR to improve anxiety‐related perioperative outcomes, however, is still in its infancy. There is little research in oncology populations, and few feasibility trials. It is essential to understand the feasibility in the targeted treatment populations [18, 19] to identify optimal timing, frequency, and tolerability of a perioperative VR intervention. This is particularly important for immersive VR experiences as most prior studies have been limited to 360° OR video tours.

Our previous work has described the development of a novel, immersive preoperative VR intervention [20]. This was developed in accordance with international methodological guidelines for medical VR for patient use where feasibility trials are emphasized as a critical component of VR development [18]. To our knowledge, this is the first study to explore the feasibility of an immersive, first‐person VR experience delivered prior to cancer surgery. The primary objective of this study was to evaluate the feasibility of this VR intervention by assessing appropriateness for inclusion based on anxiety levels, retention, tolerability, and engagement, as well as patient acceptability and impressions. Further, we examined preliminary clinical trends in perioperative state anxiety and distress, along with postoperative mental health symptomatology and pain, compared to the current standard of care (SoC). These exploratory clinical outcomes will be used as a guide to design future adequately powered randomized controlled trials, rather than evidence of clinical effectiveness.

## 2 Materials & Methods

The protocol for this single‐blind, multi‐method, randomized‐controlled feasibility trial has previously been published [20], along with a case‐series describing the first seven participants that highlighted initial feasibility to continue recruitment [21]. Follow‐up focus group results have been incorporated into the study design of this initiative [22]. The study was approved by the University of Manitoba Health Research Ethics Board (HREB#H2020:247) and registered on clinicaltrials. gov (#NCT04544618).

### 2.1 Participants

Participants were eligible if they were (a) ≥ 18 years of age, (b) able to read and speak in English, and (c) scheduled/being scheduled to undergo breast cancer surgery under general anesthesia at a large tertiary hospital in Winnipeg, Manitoba, Canada. Exclusion criteria included being unable to participate in VR (e.g., having auditory or visual impairments), or being unable to provide informed consent (e.g., having cognitive impairments). Participants were recruited using various methods (i.e., posters, preoperative education classes, surgical oncology appointments).

### 2.2 Procedure

Interested participants contacted study staff and completed telephone screening between December 2021 and December 2023. If eligible, they were randomized to either the VR intervention or the SoC group by a research team member, uninvolved with the surgery and data collection on the day of surgery. Recruitment stratification was used to ensure participants were randomized based on whether they had received neoadjuvant chemotherapy and the type of breast cancer planned (i.e., with or without reconstruction), as these factors have been reported to affect distress levels [23]. A single‐blind randomized design was applied, and participants were randomized using a web‐based random number generator.

Participants completed baseline questionnaires one to 2 weeks before surgery. The VR group completed in‐person questionnaires immediately before trialing the VR prototype, answered anxiety and distress questions during the simulation, and completed VR‐specific questions afterward. The SoC group completed one set of electronic questionnaires before surgery. All participants completed additional questionnaires on the day of surgery in the preoperative holding area and again in the OR prior to anesthetic induction, where responses were verbally provided and recorded by research staff. Follow‐up questionnaires were administered via Qualtrics (or in person if still hospitalized) at five‐ and 30‐days post‐procedure. Participants received an honorarium for participation (see Table S1 for measures and time‐points of administration).

#### 2.2.1 VR Intervention

The VR prototype [20, 21] provided an interactive simulation of the perioperative experience. An Oculus Rift S VR system (Meta Platforms) comprising two wireless hand‐held controllers and a headset connected via cable to a laptop computer were used. The participant's headset view was mirrored on the laptop along with a clinician user interface allowing personnel to help participants if needed. Using the tracked 3D positions of the controllers and headset, participants could see their virtual arms following their movements in real‐time, creating a sense of embodiment, which has been shown to increase emotional responses and might help enhance VR intervention outcomes [24]. Participants were given a minimum of 5 min to explore the interactive virtual OR (e.g., relevant equipment and machinery, sounds, personnel, mammogram on computer screen). A scripted portion followed, where participants were instructed to lie supine on the OR table and were taken through a mock anesthetic induction process by a virtual nurse and anesthesiologist. The simulation ended after the virtual oxygen mask was placed on the patient's mouth and the headset faded to black.

### 2.3 Measures

#### 2.3.1 Sociodemographics and Health Information

The sociodemographic and health questionnaire given at baseline was informed by previous research [10] (see Table 1 for variables).

**Table 1:** Baseline characteristics of participants by intervention group.

Characteristic	Total (*N* = 23) *n*, (%)	VR intervention (*n* = 12) *n*, (%)	SoC (*n* = 11) *n*, (%)
Age (mean (SD); range)	50.08 (10.16); 34–69	50.83 (10.34)	49.27 (10.38)
Marital Status			
Single	2 (8.7)	1 (8.3)	1 (9.1)
Married/Common‐law	17 (73.9)	9 (75.0)	8 (72.7)
Divorced/Separated	4 (17.4)	2 (16.7)	2 (18.2)
Highest education level			
Some high school	1 (4.3)	1 (8.3)	0 (0.0)
High school or equivalent	2 (8.7)	1 (8.3)	1 (9.1)
Some college/university	7 (30.4)	3 (25.0)	4 (36.4)
College/university degree or greater	13 (56.5)	7 (58.3)	6 (54.5)
Cancer stage			
1	3 (13.0)	2 (16.7)	1 (9.1)
2	9 (39.1)	5 (41.7)	4 (36.4)
3	2 (8.7)	0 (0.0)	2 (18.2)
Unknown (between 1 or 2)	6 (26.1)	5 (41.7)	1 (9.1)
Unknown (between 0 or 1)	3 (13.0)	0 (0.0)	3 (27.3)
Breast cancer surgery			
Lumpectomy	8 (34.7)	4 (33.3)	4 (36.4)
Single mastectomy without reconstruction	1 (4.3)	1 (8.3)	0 (0.0)
Single mastectomy with immediate reconstruction	6 (26.1)	4 (33.3)	2 (18.2)
Double mastectomy without reconstruction	1 (4.3)	1 (8.3)	0 (0.0)
Double mastectomy with immediate reconstruction	7 (30.4)	2 (16.7)	5 (45.5)
Previous surgery^a^	20 (87.0)	11 (91.7)	9 (81.8)
Anesthesia or surgery‐related issues in previous surgery^b^	4 (17.4)	3 (25.0)	1 (9.1)
APAIS cut‐off (≥ 10)	20 (87.0)	10 (83.3)	10 (90.9)
PITI cut‐off (≥ 15)	18 (78.3)	8 (66.7)	10 (90.9)
Mental health condition diagnosis^c^	11 (47.8)	7 (58.4)	4 (36.4)
Mental health support sought since cancer diagnosis	8 (34.8)	4 (33.3)	4 (36.4)
Additional cancer treatment at baseline (All chemotherapy)	8 (34.8)	4 (33.3)	4 (36.4)
Preoperative education			
Participated in a preoperative education class	16 (69.6)	8 (66.7)	8 (72.7)
Read about the surgery/recovery online	16 (69.6)	8 (66.7)	8 (72.7)
Requested additional information from healthcare provider	3 (13.0)	3 (25.0)	0 (0.0)
Spoke with others who underwent breast cancer surgery	13 (56.5)	7 (58.3)	6 (54.5)

*Note:* Independent samples *t*‐tests and chi‐square tests were used to compare groups. No significant differences emerged by intervention type.

Abbreviations: APAIS, The Amsterdam Preoperative Anxiety Information Scale; PITI, Preoperative Intrusive Thoughts Inventory; SD, Standard Deviation; SoC, Standard of Care; VR, Virtual reality.

^a^ Surgeries included (participants often mentioned multiple): caesarean section (*n* = 4), tonsillectomy (*n* = 4), lumpectomy (*n* = 2), adenoidectomy (*n* = 2), hysterectomy (*n* = 2), laparoscopy (*n* = 2), dilation and curettage (*n* = 2), trapectomy, skin cancer surgery, knee replacement, port surgery, single mastectomy with reconstruction, salpingectomy, open reduction and internal fixation, fibroid removal, deviated septum repair, carpal tunnel, hemithyroidectomy or thyroid surgery, breast cyst removal, head surgery, eye surgery, oophorectomy, lipoma removal, ear reconstruction, broken arm, appendectomy, wart removal, tubal ligation, wire guided breast biopsy.

^b^ Complications included: Premature anesthesia worn off (*n* = 2) and postoperative lung blood clot.

^c^ Mental health diagnoses included (participants often mentioned comorbid conditions): anxiety (*n* = 6), depression (*n* = 7), post‐traumatic stress disorder, obsessive compulsive disorder, substance use, clinically significant distress.

#### 2.3.2 Primary Outcomes

##### 2.3.2.1 Feasibility

Recruitment capability was assessed by recording the number of consenting individuals to all eligible participants. Retention was calculated by the proportion of individuals who completed the intervention and completed the study relative to those who consented.

##### 2.3.2.2 Tolerability

The *VR Impressions Scale* [21] assessed tolerability. Participants were asked to identify if they experienced any motion sickness during the intervention, on a scale from 0 (none) to 3 (severe). Behavioural observations of tolerability were also reported by research assistants.

##### 2.3.2.3 Acceptability

Acceptability was assessed with open‐ended questions on a text‐based survey developed by the research team. Post‐intervention, participants were asked what they liked and disliked about the intervention, and at 5 days post‐surgery they were additionally asked if they found the VR helpful, how they thought it impacted their surgery and recovery, and if they had any other suggestions for improvement (open‐ended). Additionally, participants were given 11 statements about their VR experience (See Figure 2 for statements) and were asked to rate these statements from 0% (completely disagree) to 100% (completely agree). Participants were also asked if the intervention was worthwhile considering the time commitment of the VR program and questionnaire completion (Yes/No) [21, 25]. Participants were asked about additional elements they wished had been included in the intervention from a series of options and could provide suggestions to inform future development.

##### 2.3.2.4 Engagement

*The iGroup Presence Questionnaire*, a 14‐item valid and reliable questionnaire, measured the sense of VR presence (i.e., the sense of *being* in the VR environment) [26]. This questionnaire was given post VR‐intervention, and participants self‐rate their agreement with each statement on a 7‐point Likert Scale, ranging from ‐3 (not consistent) to 3 (very consistent; e.g., “How much did your experience in the virtual environment seem consistent with your real‐world experience?”). There are three subscales: spatial presence (i.e., sense of being physically present in the virtual environment [VE]), involvement (i.e., measuring attention devoted to the VE and involvement experiences), and experienced realism (i.e., measuring subjective experience of realism in the VE). A single item assesses presence. Scores are recoded and averaged, with total scores ranging from 0 to 6. Higher scores represent greater presence in the VE. Though no established cut‐offs exist, research suggests scores of ≥ 3.86, ≥ 4.50, ≥ 4.00, and ≥ 3.38 are acceptable for presence, spatial presence, involvement, and experienced realism, respectively [27].

#### 2.3.3 Pilot Outcomes

##### 2.3.3.1 Preoperative Anxiety

*The Amsterdam Preoperative Anxiety and Information Scale* (APAIS) is a 6‐item measure used to assess patient's anxiety and information requirement in the preoperative phase [28], and the *Pre‐operative Intrusive Thoughts Inventory* (PITI) is a 20‐item measure that assesses sources of preoperative anxiety [29]. Scores of ≥ 10 and ≥ 15 are indicative of clinically significant preoperative anxiety on the APAIS and PITI, respectively [28, 29]. Both measures have strong psychometric properties and higher scores indicate greater preoperative anxiety [28, 29]. These measures were provided both at baseline and again in the preoperative holding area.

##### 2.3.3.2 State Anxiety and Distress

Two visual analogue scales, the *NCCN Distress Thermometer* and an author‐adapted *Anxiety Thermometer*, were used to measure preoperative distress and anxiety, at the following perioperative time points: baseline, day of surgery before entering the OR, in the OR immediately before anesthetic induction, and 5 days post‐surgery [21, 30]. Participants were asked to rate their past‐week distress and anxiety on a scale from 0 (no distress/anxiety) to 10 (extreme distress/anxiety), and this was adapted in the OR to assess momentary anxiety and distress. Higher scores indicate greater distress and anxiety. *The Distress Thermometer* has been validated in oncological samples [31].

At the 5‐day postoperative assessment, participants were asked which of those four anxiety measures best captured their perioperative mental health experiences to inform the future trial.

#### 2.3.4 Exploratory Outcomes

##### 2.3.4.1 Generalized Anxiety and Depressive Symptoms

Generalized anxiety and depressive symptoms were measured using the short‐form *Patient‐Reported Outcomes Measurement Information System (PROMIS)* Anxiety and Depression Scales. There are 8 items in each measure and higher scores on these self‐report measures indicate a greater amount of the construct being measured. The PROMIS measures are based on item‐bank items with strong psychometric properties [32]. These measures were provided at baseline and 30 days post‐surgery.

##### 2.3.4.2 Pain Intensity

Pain intensity was measured with the *PROMIS Pain Intensity–Short Form*, that uses 3 items and assesses intensity of pain, on a scale from 1 (had no pain) to 5 (very severe) [33]. Participants received this questionnaire on postoperative days 5 and 30.

### 2.4 Analyses

#### 2.4.1 Quantitative Analysis

Sample characteristics, baseline data, recruitment rates (including reasons for non‐participation), and acceptability outcomes were described using descriptive statistics. Independent samples *t*‐tests and chi‐square tests compared demographic differences between intervention and control groups. Repeated measures ANOVAs were used to assess changes in participant self‐reported symptoms across the study. In cases where Mauchly's Test of Sphericity was significant (e.g., repeated measures ANOVA for distress and anxiety), an appropriate correction was used (Greenhouse‐Geisser). Due to the small sample size, findings are presented with additional line graphs to illustrate trends. Quantitative analyses were conducted in SPSS (V29.0).

#### 2.4.2 Qualitative Analysis

All open‐ended responses were compiled in Excel, and data were analyzed using qualitative inductive content analysis [34]. Two coders trained and experienced in qualitative inductive content analysis (GL and KP) began by closely reviewing the data to familiarize and prepare for coding. Staying close to the data, both coders independently analyzed the raw data by small unit (e.g., paragraph), labeling each small unit with meaningful identifiers. Coders met to review small meaning units and discussed ways to categorize data into broader categories. Coding was completed iteratively, moving back and forth between the raw data and drafted thematic categories. The thematic categories were further refined with the addition of more data and coding discussions. Any discrepancies in coding were resolved through collaborative discussion and consultation with co‐authors, who held unique positions in response to the research, including as graduate students, qualitative and quantitative researchers, and medical experts. A rigorous approach to qualitative analysis was completed by following several approaches outlined in Tracy's (2010) Criteria for Excellent Qualitative Research [35] (e.g., credibility is demonstrated by triangulation [i.e., multiple researchers in different disciplines analyzing data]).

### 2.5 Deviations to the Protocol

There were several adjustments to the published protocol (described there) [20]. For example, we had initially recruited for a third group (*N* = 45, *n* = 15 in each group; in addition to the intervention and SoC), an active control VR group (*Nature Treks*), however, slow recruitment related to COVID‐19 necessitated the removal of the *Nature Treks* group.

## 3 Results

### 3.1 Recruitment and Retention

**Figure 1:**
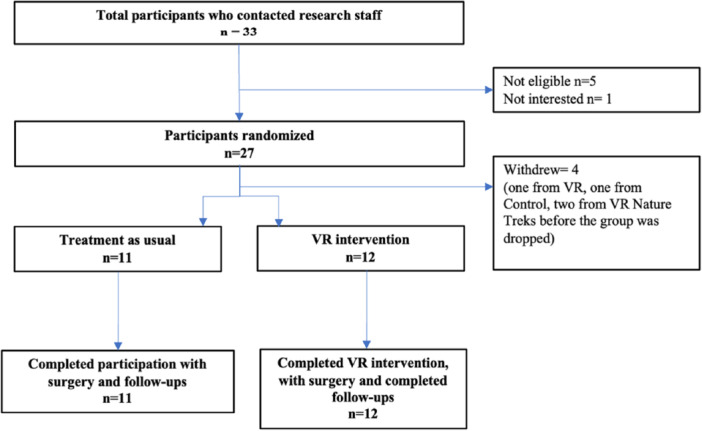
Participant flowchart diagram. VR = Virtual reality.

Thirty‐three individuals with breast cancer expressed interest in participating. Five were ineligible because their surgeries had either already occurred or were scheduled within 1 week. One participant was no longer interested. Twenty‐seven participants (81.8%) were then randomized to either the VR OR intervention group or the SoC group (Figure 1). Four participants withdrew before completing the intervention (one from the SoC group, one from the VR group, and two from the VR active control [Nature Treks] condition that was eliminated; the latter two had not been re‐randomized). Participants withdrew because they were overwhelmed (*n* = 2; e.g., with medical appointments), lost interest (*n* = 1), or did not having enough time (*n* = 1). This resulted in a final sample of *N* = 23 (85.2%) completing the study (*n* = 11 SoC [91.7%]; *n* = 12 VR [92.3%]). All remaining participants completed the full study, with no participants lost to follow‐up.

### 3.2 Participant Characteristics

Participants were on average 50.1 years old (*SD* = 10.2; range 34–69 years). Participants all identified as female, and the largest proportion were undergoing a form of mastectomy (65.2%), and 87.0% had undergone a previous surgery. The majority (69.6%) had attended an optional preoperative education class about their upcoming surgery, read about the surgery/recovery process online (69.6%), and spoke with someone who underwent breast cancer surgery (56.5%). At baseline, between 78.3% and 87.0% had clinically significant preoperative anxiety on the PITI and the APAIS, respectively. Most participants (69.6%) felt that the PITI best captured their preoperative anxiety experiences. No significant differences emerged between the two groups on any baseline characteristics, though trends suggested that the SoC group had elevated baseline preoperative anxiety (90.9% of the SoC group met criteria for clinically significant anxiety on the PITI compared to 66.7% of the VR group). Other characteristics are shown in Table 1.

### 3.3 Engagement

On average, participants spent 10 min 37 s (SD = 1.29) engaged in the VR. Presence (*M* = 4.36, SD = 1.21, range 2–6) and experienced realism scores (*M* = 3.39, SD = 0.55, range 2.25–4.25) were acceptable. Spatial presence (*M* = 4.11, SD = 0.83, range 3.20–6.00) was within the marginally acceptable range, and the average involvement score (*M* = 3.02, *SD* = 1.09, range 1.25–4.25) was below acceptable thresholds [27].

### 3.4 Retention and Tolerability

All participants completed the VR, and all questionnaires across time points. No participants reported experiencing motion sickness during the intervention, or any other adverse outcomes.

### 3.5 Acceptability

#### 3.5.1 Quantitative Results

**Figure 2:**
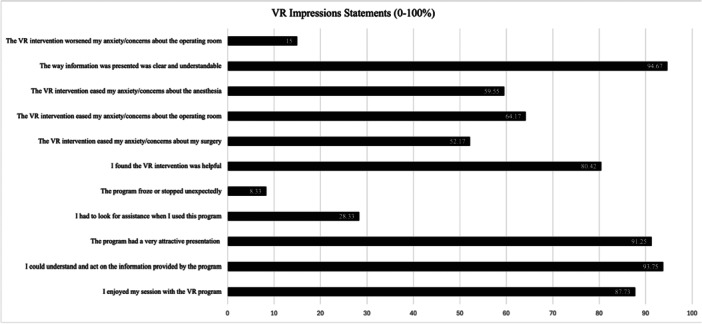
Quantitative VR impressions. *Note: n* = 12. VR = Virtual reality.

*The VR Impressions Scale* demonstrated that most participants enjoyed the VR (87.7%), felt that the program presentation was attractive (91.3%), and found it helpful (80.4%). Many participants indicated that the use of VR resulted in easing anxiety/concerns about anesthesia (59.6%), their surgery (52.2%), and the OR (64.2%; Figure 2). Less than a third required assistance or had technical issues.

All participants indicated that the intervention was worthwhile relative to the time commitment. Participants indicated what additions to the prototype would strengthen the VR experience, first from a provided list of possibilities and then in response to an open‐ended question. From the list, 58.3% would have liked a feature of being wheeled into the OR, 33.0% would have liked a chance to ask the virtual anesthetist or nurse questions about their surgery, 25.0% would have liked to have the opportunity to try on actual equipment (e.g., oxygen mask) while engaged in the simulation and 25.0% would have liked the opportunity to learn about the various OR machines. Other suggestions (open‐ended) included seeing the actual surgery itself, putting on the finger pulse oximeter or blood pressure cuff during simulation, having music playing, and seeing the real‐time pulse/heart rate.

#### 3.5.2 Qualitative Results

Six main themes emerged from the open‐ended responses: Realism, Interactivity, Preparation for the OR, Psychological and Emotional Impact, Technical Issues, and Supporting Others (Table S2).

In the category of realism, individuals described the graphics of the VR intervention as both unrealistic (“Was not at all like the actual surgical experience”) and realistic (e.g., “It was very good, very real to life.”). Another theme was interactivity, where more participants stated that the intervention needed enhanced interactivity (e.g., “Needs to be more interactive. There was a lot of dead space just observing around the OR”), while one participant highlighted that the technology was engaging (e.g., “it was interactive and could play a bit with it”).

Preparation for the OR was the most frequently coded theme. Here, most participants discussed that the VR intervention prepared them for what to expect in the OR (e.g., “provided an experience of what I should expect to happen for my surgery”). Some described it as unhelpful, as they had already experienced surgery before (e.g., “It didn't help me very much as I've had 3 previous surgeries”). However, most of these individuals who did not find it helpful noted that it would be beneficial for people who have never been in an OR before (e.g., “I've had surgery before so this wasn't new for me. Would be great for first timer.”).

The main theme of psychological and emotional impact encompassed participants describing how the VR intervention bolstered their emotions or mindset for surgery. The majority described feeling more relaxed (e.g., “you feel more relaxed for the surgery”), having increased awareness or mindfulness (e.g., “made me realize how I feel about surgery”) or having eased anxiety and fears (e.g., “I thought it was a good way to help calm some of my fears”). One person described adverse emotional impacts (“It actually gave me more anxiety leading up to the surgery date”).

Technical issues were noted by a few participants, mainly to do with calibration (i.e., orientation) issues (e.g., “the program had to be reset” several times) and technology crashes. Finally, two participants expressed that participating in the intervention was important to them because it allowed them to help others.

### 3.6 Pilot Outcomes

#### 3.6.1 Preoperative Distress and Anxiety

**Figure 3:**
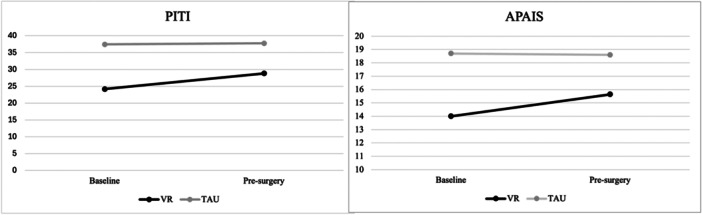
Means across time for preoperative anxiety on the APAIS and PITI. APAIS = The Amsterdam Preoperative Anxiety Information Scale, PITI = Preoperative Intrusive Thoughts Inventory, TAU = Treatment as Usual, VR = Virtual Reality.

Neither the main effect of time, nor time*intervention was significant for the APAIS (*F*(1,19) = 1.22, *p* = 0.28) or PITI (*F*(1,18) = 1.43, *p* = 0.25; Figure 3) from baseline to pre‐surgery.

#### 3.6.2 Perioperative State Distress and Anxiety

Participants in the VR group reported an average of 3.30 (*SD* = 3.20) for anxiety and an average of 3.10 (*SD* = 3.03) for distress mid‐way through the intervention on the thermometers, indicative of non‐clinically significant distress and anxiety.

**Figure 4:**
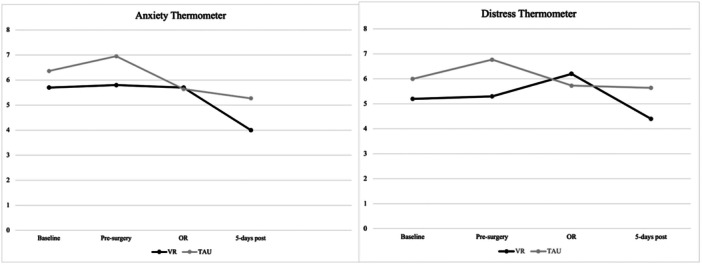
Means across time for preoperative anxiety on the distress and anxiety thermometers. OR = Operating Room, TAU = Treatment as Usual, VR = Virtual Reality.

There were no significant differences in distress on the *Distress Thermometer* over time, and time*intervention was not significant either (*F*(2.01,38.16) = 0.96, *p* = 0.39). Similarly, on the *Anxiety Thermometer*, anxiety significantly decreased across time (*F*(3,57) = 3.17, *p* < 0.05), though, time*intervention differences were not significant (*F*(3,57) = 0.52, *p* = 0.67; Figure 4). Trends saw a greater decrease in distress postoperatively for the VR group as compared to the SoC group, though a greater increase in distress in the OR. Similarly, there was a trend of lower anxiety for the VR group at 5 days post‐surgery, compared to the SoC group.

### 3.7 Exploratory Outcomes

There were no significant group differences in changes from baseline to 30 days post‐surgery for anxiety (*F*(1,21) = 0.59, *p* = 0.81) and depressive (*F*(1,21) = 0.85, *p* = 0.37) symptoms. Independent samples *t*‐tests also did not reveal significant differences between groups on pain intensity at 5 days (*t*(21) = −0.29, *p* = 0.39) or 30 days (*t*(21) = 0.80, *p* = 0.47) post‐surgery.

## 4 Discussion

To our knowledge, this represents the first perioperative VR exposure‐based randomized‐controlled trial in North America and the first to examine its utility for oncological surgery. Because this was designed as a pilot feasibility trial, the findings of this study should be interpreted with the understanding that the study was not powered to detect statistically significant clinical effects. The findings within the current study, coupled with the findings from follow‐up focus groups completed with a sub‐sample of these participants [22, 36], demonstrate the current VR program as an acceptable and feasible preoperative intervention for patients undergoing breast cancer surgery. More than three‐quarters of participants indicated clinically significant levels of preoperative anxiety, underscoring the importance of targeted interventions in this vulnerable population. Most participants in the VR group found it helpful in easing some of their anxiety/concerns about the surgery, the OR, and the anesthesia, even in its preliminary form. There were also no adverse effects reported, and the intervention was completed in the window between the surgical consultation and the OR without difficulty, highlighting feasibility. Although the current study was not powered to detect statistically significant clinical outcomes, some positive (but non‐significant) trends for the intervention group on perioperative anxiety and distress emerged (assessed from baseline to 5 days postop), particularly in the postoperative period. These exploratory trends support the need to evaluate this intervention in a larger, adequately powered, randomized trial. With further development of the intervention, it has the potential to make a noticeable impact for a large proportion of patients with preoperative anxiety and distress. Indeed, all participants in the current study emphasized that the intervention, even in its prototype form, was worthwhile considering the time commitment.

In terms of procedural feasibility, recruitment was overall low. The multi‐method recruitment strategy did not allow us to determine the total number of eligible participants from the larger population, but we estimate given the large recruitment timeframe (2 years), there were possibly a couple of hundred eligible participants. However, we believe this difficulty in recruitment was partly related to the impact of the COVID‐19 pandemic [20], which may have deterred people from participating in non‐mandatory appointments. Despite the low recruitment rate, retention rates were promising. Of the 28 individuals who expressed interest and were eligible, 82.1% were randomized and completed the study, well above the recommended rates for recruitment in feasibility study best practice guidelines [37]. Future research should focus on optimizing initial recruitment from the larger cancer surgery population. In terms of VR program engagement, results demonstrated acceptable rates of realism and presence, suggesting participants found it an immersive experience. Results, however, indicated lower levels of involvement. This finding is corroborated by qualitative findings showing that some participants described some difficulty interacting with the program. Future iterations of the program should therefore incorporate gamified, interactive elements to enhance overall engagement, as these approaches have demonstrated positive effects on mental health symptomology [38] and show high potential for anesthesia‐related care [39].

When developing VR interventions, best practice guidelines recommend completion of feasibility trials integrating patient input into the intervention prior to finalizing and testing its impact in larger trials [18]. Although the VR intervention was acceptable to patients, some limitations should be the focus of future research. Although most participants reported the VR as acceptable and helpful, one individual noted increased anxiety. This may relate to the brief duration of the simulation; systematic and prolonged exposure is typically required for habituation and anxiety reduction [40]. Thus, the short exposure may have been insufficient for those with high baseline anxiety, and future iterations should allow adequate, and possibly repeated, exposure. Supporting this, our prior work with six participants using the study's biofeedback component showed increased arousal during the simulation [36]. In this trial, midpoint anxiety and distress were generally low, but scores ranged from 0 to 10 and 0 to 8, indicating that some found the VR highly anxiety‐provoking. Individual variability in required habituation dosage may explain this response. Additionally, reactions may have reflected VR use itself rather than the program content, suggesting the value of including a sham VR control group in future research.

The majority of participants (69.6%) had attended a preoperative preparation course. Preoperative information sessions have been shown to decrease preoperative anxiety in cancer surgery patients [41, 42]. Participation in these sessions may have contributed to reductions in anxiety and distress as well among the sample. However, attendance rates were similar and appeared balanced between the VR and SoC groups, indicating that any effect these had on anxiety and distress would likely have been balanced across groups and would not explain any trend differences in outcomes. Unfortunately, their overall impact cannot be assessed as we did not assess whether participation in these classes was associated with reduced anxiety. However, we know that these classes do not specifically target anxiety. Similarly, while there were no significant differences across groups, 87.0% of participants of total participants had undergone a surgery before, and qualitative feedback from participants highlighted the utility of the VR intervention for those undergoing their first OR surgery [22]. This may have impacted null effects for pilot outcomes.

### 4.1 Real‐World Implementation and Applicability

Despite the high prevalence of preoperative anxiety identified in this study and others, access to empirically‐supported psychological treatments before surgery remains limited. This relates to both the short timeframe before surgery, as well as the limited access to mental health professionals to deliver such interventions to the large and growing numbers of surgical patients. As such, interventions that can be delivered in a brief format and scaled to multiple patients, such as VR‐based programs, may offer an important solution to perioperative mental health care. As demonstrated in this trial, this intervention was implemented without disrupting routine clinical care or being affected by routine clinical activities, both of which have been identified as barriers in previous VR feasibility studies addressing preoperative anxiety e.g., [42, 43]. This highlights the feasibility of incorporating this intervention into existing perioperative standards of care. Furthermore, the programs flexibility allows a broader clinical implementation, as it could be delivered in the comfort of a patient's own home or it could be integrated into pre‐anesthesia clinic appointments. Importantly, although the study focused on breast cancer patients, the core elements of the intervention are also broadly applicable to other surgeries performed under general anesthesia.

### 4.2 Study Limitations

The results of this study should be interpreted considering several limitations. The sample size was appropriate for a feasibility trial but not powered to detect clinically significant statistical effects; therefore, pilot results should be interpreted with caution and should be used as a guide to design future adequately powered RCTs, rather than evidence of clinical effectiveness. We lacked demographic and other information for individuals who dropped out before completing baseline questionnaires, limiting understanding of recruitment and attrition. Although not statistically different, the VR group appeared to have lower baseline preoperative anxiety, which may have influenced findings; larger samples that can stratify by baseline anxiety levels are needed and will be important to more accurately isolate the intervention effects. Some postoperative questionnaires were completed 5 days after surgery, raising the possibility of recall bias in reports about the VR intervention. Finally, the sample primarily consisted of married/partnered, educated women, limiting generalizability to more diverse populations and potentially affecting feasibility outcomes.

## 5 Conclusions

This study has implications for the growing literature examining VR interventions, a novel and potentially accessible treatment modality, targeting mental health during the perioperative period. Over 75% of participants in this study had clinically significant levels of preoperative anxiety, and prior research indicates that perioperative anxiety is the worst reported component of the surgical experience, emphasizing the need to better support mental health in surgical populations. Patient feedback from this study and others has directly informed the development of a vastly expanded VR intervention. This developed VR intervention will be used in the most comprehensive perioperative VR study to date, which aims to improve perioperative mental health and its adverse health sequelae for patients undergoing cancer surgery. This and other VR research should aim to not only examine the impact of VR interventions on patient‐reported outcomes including anxiety but also other downstream postoperative health sequelae such as such as postoperative complications, readmissions, and length of stay.

## Conflicts of Interest

The authors declare no conflicts of interest.

## Protocol

The protocol citation is: El‐Gabalawy R, Sommer JL, Hebbard P, Reynolds K, Logan GS, Smith MSD, et al. An Immersive Virtual Reality Intervention for Preoperative Anxiety and Distress Among Adults Undergoing Oncological Surgery: Protocol for a 3‐Phase Development and Feasibility Trial. JMIR Res Protoc. 2024 May 14;13:e55692.

## Synopsis

Virtual reality (VR) shows promise as a tool for reducing preoperative anxiety, a factor linked to poor postoperative outcomes. A novel VR designed to familiarize patients with hospital environments was found to be acceptable and feasible by participants. Preliminary trends suggest that VR may help reduce anxiety and distress perioperatively.

## Supporting information

Supporting File

Supporting File

Supporting File

## Data Availability

The data that support the findings of this study are available from the corresponding author upon reasonable request.
