# Supplementary material for: An Interactive Preoperative Virtual Reality Intervention for Breast Cancer Patients Undergoing Oncological Surgery: A Feasibility and Pilot Randomized Clinical Trial
Source: J Surg Oncol. 2026 Apr 23;134(1):5–15. doi: 10.1002/jso.70269 (PMC13378775; doi:10.1002/jso.70269)
Supplement: Supplementary file 1 — Supporting File [file JSO-134-5-s002.docx]

|  | Baseline questionnaires (1 to 2 weeks pre-surgery) | Post-VR questionnaires (VR group only; immediately after VR trial) | Day of surgery before the OR | Day of surgery in OR before anesthetic induction | 5 days post-op | 30-days post-op |
| --- | --- | --- | --- | --- | --- | --- |
| APAIS | X |  | X |  |  |  |
| PITI | X |  | X |  |  |  |
| NCCN Distress Thermometer and Anxiety Thermometer | X |  | X | X | X |  |
| PROMIS Anxiety and Depression Scales | X |  |  |  |  | X |
| PROMIS Pain intensity Scale |  |  |  |  | X | X |
| VR Impressions Scale |  | X |  |  |  |  |
| The iGroup Presence Questionnaire |  | X |  |  |  |  |

**Supplemental Table 1**. *Questionnaires Administered at Different Time Points.*

Note. APAIS = The Amsterdam Preoperative Anxiety and Information Scale; OR = Operating Room; PITI = Pre-operative Intrusive Thoughts Inventory (PITI); PROMIS = Patient-Reported Outcomes Measurement Information System*;* VR = Virtual reality.
