# Supplementary material for: An Interactive Preoperative Virtual Reality Intervention for Breast Cancer Patients Undergoing Oncological Surgery: A Feasibility and Pilot Randomized Clinical Trial
Source: J Surg Oncol. 2026 Apr 23;134(1):5–15. doi: 10.1002/jso.70269 (PMC13378775; doi:10.1002/jso.70269)
Supplement: Supplementary file 2 — Supporting File [file JSO-134-5-s001.docx]

**Supplemental Table 2**

*Examples of Supporting Quotes for Themes that Emerged Via Open-Ended Questions*

| Theme | Example quotes |
| --- | --- |
| Realism | “it was realistic to my past surgical experiences”  “Was not at all like the actual surgical experience” |
| Preparation for OR | **If you found it helpful: In what ways was the VR intervention helpful?**  “The sounds and sights and procedures give you a test run- prepares you for the actual day”  **What did you like about the VR intervention?**  “just getting the feel of an OR”  **“**Seeing the space and how the steps would go to be put under”  “it is a great tool for someone who has never had a surgery before”  “It was good but more for people who have not had surgery” |
| Interactivity | “Needs to be more interactive. There was a lot of dead space just observing around the O.R.”  “it was interactive and could play a bit with it”  “It might help if someone was actually putting on the finger reader or blood pressure cuff to make it more realistic” |
| Psychological and emotional impact | “you feel more relaxed for the surgery”  “think I can relax a bit now when its time for me to have my surgery”  “Made me aware of how I feel at the moment. Made me aware of my fears and worries prior to surgery.”  “I was reassured having seen the room and experience of the IV and mask and going to sleep from that POV. Having never had surgery, I thought that that would be my moment of panic, but that never came and I think it was in small part because it was all as expected from the VR.”  “It actually gave me more anxiety leading up to the surgery date” |
| Technical issues | Head set didn't work well  yeah, sadly the program had to be reset a bunch of times bc it thought I was standing when I was sitting and lying down it thought I was sitting and the orientation was off, it did not recognize I was lying down bc I would have had to lie down into the wall. it brought me out of it |
| Supporting others | **Please briefly explain why it was or was not worthwhile:**  Helping others  I like to help with research and I'm curious about VR and MH initiatives |

*N* = 11. OR = Operating Room.
